# Successful laparoscopic management of diaphragmatic pregnancy:a rare case report and brief review of literature

**DOI:** 10.1186/s12884-019-2248-0

**Published:** 2019-03-28

**Authors:** Lifeng Chen, Jinwei Liu, Jing Shu, Wenjie Zeng, Xiaofeng Zhao

**Affiliations:** Departments of Gynecology, Zhejiang Provincial People’s Hospital, People’s Hospital Of Hangzhou Medical College, No. 158 Shangtang Rd, Hangzhou, 310014 People’s Republic of China

**Keywords:** Abdominal ectopic pregnancies, Diaphragmatic, Pain

## Abstract

**Background:**

Abdominal ectopic pregnancies are rare category of ectopic pregnancies.We describe a ectopic pregnancy implanted on the surface of diaphragm and highlight the difficult clinical course of an abdominal ectopic pregnancy managed with strategic procedural intervention.

**Case presentation:**

A 33-year-old Chinese woman with abdominal pain was diagnosed with ectopic pregnancy implanted in the liver according to CT and advised to transfer the patient to tertiary referral center. The patient had a significant increasing abdominal pain and intense reflex sensitivity in the right shoulder when she presented to our hospital. Laparoscopic surgery was decided immediately with the impression of ruptured abdominal pregnancy and hemorrhagic shock. It was found that ectopic pregnancy was implanted on the diaphragmatic surface.The pregnant tissue was completely resected from the diaphragm and bipolar electrocoagulation was used to control bleeding. The patient was stable and discharged 3 days after surgery in a good condition.

**Conclusion:**

CT or MRI should be considered as an alternative to TVS in the management of ectopic pregnancy patients with unusual presentations. A thorough observation of the entire pelvis and upper abdomen during laparoscopic exploration is crucial for diagnosis.

## Background

Abdominal ectopic pregnancies are rare category of ectopic pregnancies, which occur most frequently in the douglas of pouch, omentum and organs such as liver, spleen and bowel and large vessels [1–4] are associated with higher morbidity due to their late presentation and diagnostic difficulties [5–8]. A PUBMED search in the English language from 1976 to January 2016 using search terms “ectopic”, “pregnancy or gestation”, and “Non-tubal” or “abdominal”or “abdominal”,several published reports are found but few studies reported cases of ectopic pregnancy implanted in the surface of diaphragm. We describe an unusual case that highlights the difficult clinical course of an abdominal ectopic pregnancy managed with strategic procedural intervention. Written informed consent was obtained from the patient. We also present a brief review of the literature on this rare event.

## Case presentation

**Fig. 1:**
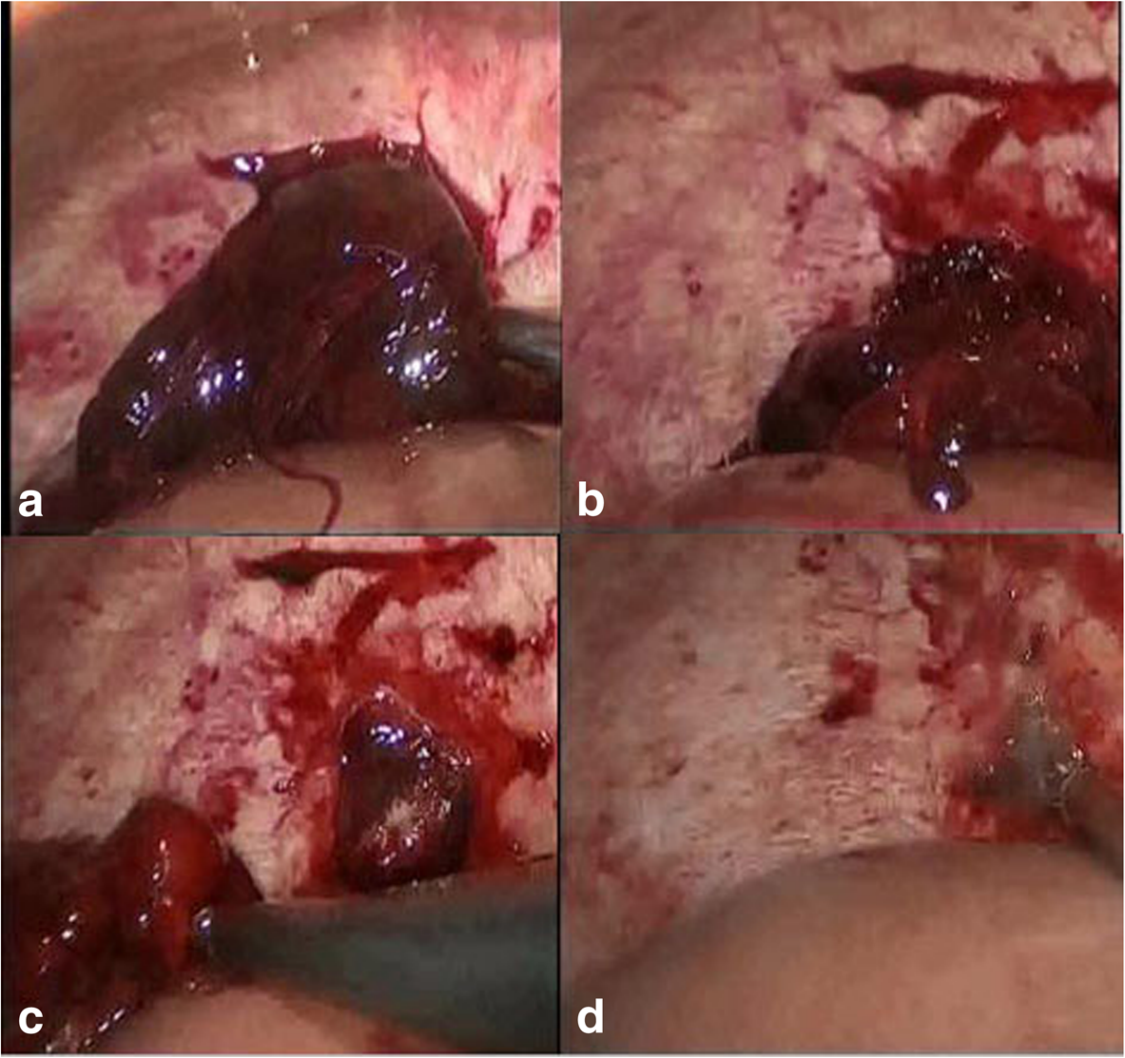
**a**: A mass of 80*50 mm on the surface of diaphragm. **b**: Bleeding occurred at the attachment site. **c**: Villus tissues was found invade the diaphragm. **d**: A bipolar electrocoagulation was placed to achieve hemostasis

**Fig. 2:**
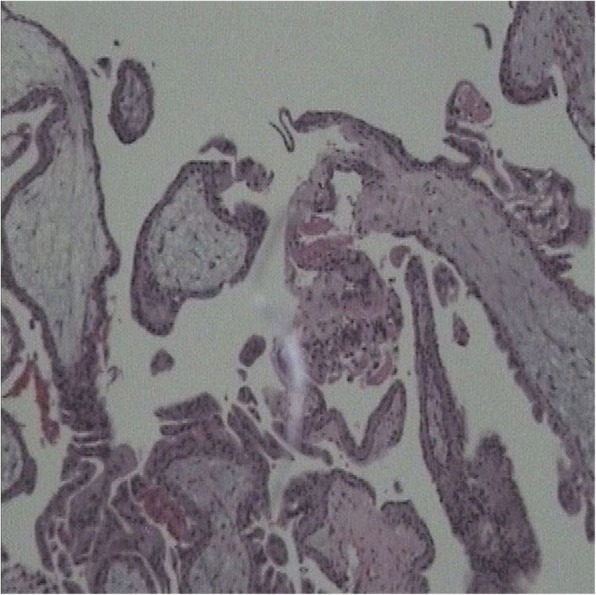
Histopathological findings of a ectopic pregnancy. Chorionic villi along withe a blood clot are evident

A 33-year-old chinese woman with a history of previous cesarean section was referred to her local emergency room with 8 weeks’ delay of menstruation and frequent increasing pain in the right upper quadrant of her abdomen and intense reflex sensitivity in the right shoulder for a duration of one day. In her medical history there was no record of use of an IUD, endometriosis, pelvic inflammatory disease, tubal surgery, intrauterine device, or previous ectopic pregnancy. Laboratory evaluation showed quantitative hCG of 3129.94 IU/L and hemoglobin of 10.3 g/dL. TVS examination demonstrated no evidence of intrauterine pregnancy, a normal bilateral adnexa and a large amount of free fluid in the abdomen. Considering the above factors, a CT scan of the abdomen and pelvis was performed. A 90-mm-long mixed hypodense mass was evident on the upper surface of the right liver lobe. She was diagnosed with ectopic pregnancy implanted in the liver and advised to transfer the patient to tertiary referral center. The patient traveled to our hospital and presented to the emergency room with increasing abdominal pain and weakness. On examination, her pulse was 109beats/minute and blood pressure was 90/50 mmHg. Diagnostic abdominocentesis was performed and found blood uncoagulable. Given the concern for ruptured abdominal pregnancy and hemorrhagic shock, she was taken laparoscopic surgery immediately. The laparoscopy revealed a significant hemoperitoneum (of approximately 1500 ml of blood). The uterus and ovaries appeared normal, with a corpus luteum cyst in 50 mm on the left ovary. There was no evidence of bleeding from the pelvic organs while a mount of free blood appearred aroundding perihepatic and spleen. The upper abdomen was inspected and a active bleeding was discovered from the mass with the size of 80 mm* 50 mm on the surface of diaphragm (Fig. 1). The mass was completely resected from the diaphragm. When villus tissues invade the diaphragm, bleeding occurred at the attachment site, a bipolar electrocoagulation was placed to achieve hemostasis. The patient was stable and discharged 3 days after surgery with instruction for making follow-up weekly. The pathology of 7 days after surgery confirmed ectopic pregnancy (Fig. 2). Her hCG levels returned to the normal range in two weeks.

## Discussion

Ectopic pregnancy with risks of major haemorrhage is one of the leading direct causes of maternal mortality. Nearly all ectopic pregnancies (97%) are implanted in the fallopian tube, while 0.9–1.4% of ectopic pregnancies are implanted in the abdominal cavity [9]. It has been reported [4, 10, 11] that there are two types of abdominal ectopic pregnancies: Primary and secondary, the former happened when the fimbrial end does not ‘pick up’ the ovulated follicle,while the latter is related to tubal abortion via the fimbria and peritoneal implantation1. Risk factors of the two including pelvic inflammatory disease, pelvic surgery history, use of certain types of IUD, a previous ectopic pregnancy, and older maternal age and as sisted conception. The clinical presentation is variable, including elevated quantitative hCG, acute abdomen pain, painful foetal movements, BPV bleeding or(with massive hemorrhagic),nausea and vomiting. So,early and timely diagnosis is critical for successful treatment with regards to severity of morbidity and mortality risk. The consistent clinical presentation of our patient was abdominal pain.

Ectopic pregnancy should be considered in all reproductive-age females presenting with abdominal pain or vaginal bleeding. If the patient is stable, the diagnosis is based on the detection of the dynamic changing of beta subgroup of hCG in blood as well as on TVS and physical examination findings [12]. In patients with positive hCG and initial TVS, diagnosis can be straightforward [13]. While for patients with positive hCG and initial negativet TVS, repeat TVS 2–7 days from initial presentation is advised. if the follow-up TVS is inconclusive for pelvic ectopic, then an a diagnostic laparoscopy may be considered. Isolated case reports CT been used for diagnosis of splenic pregnancy [14, 15],while ultrasound alone reportedly misses > 50% of abdominal pregnancies.

According to the literature, more than 1% of ectopic pregnancies are associated with undetectable levels of hCG [16]. Daniilidis A [17] performed a brief review of the literature for ectopic pregnant patient with negative pregnancy test, and proposed that it is a considerable challenge to identify a patient with an ectopic pregnancy at risk of rupture. For these cases, undertaking careful clinical examination and TVS of the patient play an important role in the diagnosis. However, for the hemodynamically unstable patient, timely laparoscopy exploration with advantage of better vision can be performed to identify and treat the site of ectopic implantation. For our patient, the combination of CT scan and hCG examination is sufficient to diagnose abdominal ectopic pregnancy.

There is no standard on the optimal management of abdominal pregnancy. Management approaches reported in the literature vary from medical treatments to surgery. Medical therapy with MTX has been reported successful for unruptured abdominal pregnancy [15, 18]. However, the surgical exploration should be done if hCG over 5000 IU/L and fetal cardiac activity, both of which are most common in splenic pregnancies.Emergent laparotomy and splenectomy is advised for splenic pregnancies [19],thought a few of studies reported MTX injection is effective for them [20–22].The most serious complication for abdominal pregnancy is hemorrhage from the placental site. Operative laparoscopy has been confirmed safe and sustainable in most women with hemodynamic instability [23]. Most literatures proposed that the placental insertion site should be left undisturbed in live or spleen found during the surgery and methotrexate need used as further therapy [24]. For our case, the early diagnosis in unruptured ectopic pregnancy in upper abdominal base on a combination of CT scan and the positive of serum hCG. CT may be able to provide more value for the diagnosis than TVS. During the surgery, a complete upper and lower abdominal survey was done and the villus tissues were removed by elastic separating plier and the bleeding site was treated by bipolar electrocoagulation. The patient was recover with no further complaints or complications. Even after the decision of surgery intervention, individual therapy are administrated for abdominal pregnancy. In our case, the pregnancy tissue was implanted in the surface of diaphragm with poorer vascular than liver or spleen, complete removal for pregnancy tissue thus is performed.

## Conclusions

CT or MRI could be another choice instead of TVS for the difficult patient. A thorough observation of the entire pelvis and upper abdomen during laparoscopic exploration is crucial for diagnosis. It is important to adequately assess the effectiveness and safety of management choice.

## Abbreviations

CT: Computed tomography scan
hCG: Human chorionic gonadotrophin
IUD: Intrauterine contraceptive devices
MRI: Magnetic resonance imaging
PV: Bleeding per vaginum
TVS: Transvaginal ultrasound
